# Relay of Herpes Simplex Virus between Langerhans Cells and Dermal Dendritic Cells in Human Skin

**DOI:** 10.1371/journal.ppat.1004812

**Published:** 2015-04-13

**Authors:** Min Kim, Naomi R. Truong, Virginia James, Lidija Bosnjak, Kerrie J. Sandgren, Andrew N. Harman, Najla Nasr, Kirstie M. Bertram, Norman Olbourne, Shailandra Sawleshwarkar, Kaylene McKinnon, Ralph C. Cohen, Anthony L. Cunningham

**Affiliations:** 1 Centre for Virus Research, Westmead Millennium Institute, Sydney, Australia; 2 Sydney Medical School, University of Sydney, Sydney, Australia; 3 Sydney Institute of Plastic and Reconstructive Surgery, Sydney, Australia; 4 Western Sydney Sexual Health Centre, Sydney, Australia; 5 Department of Surgery, Children’s Hospital at Westmead, Sydney, Australia; Tulane Health Sciences Center, UNITED STATES

## Abstract

The mechanism by which immunity to Herpes Simplex Virus (HSV) is initiated is not completely defined. HSV initially infects mucosal epidermis prior to entering nerve endings. In mice, epidermal Langerhans cells (LCs) are the first dendritic cells (DCs) to encounter HSV, but it is CD103^+^ dermal DCs that carry viral antigen to lymph nodes for antigen presentation, suggesting DC cross-talk in skin. In this study, we compared topically HSV-1 infected human foreskin explants with biopsies of initial human genital herpes lesions to show LCs are initially infected then emigrate into the dermis. Here, LCs bearing markers of maturation and apoptosis formed large cell clusters with BDCA3^+^ dermal DCs (thought to be equivalent to murine CD103^+^ dermal DCs) and DC-SIGN^+^ DCs/macrophages. HSV-expressing LC fragments were observed inside the dermal DCs/macrophages and the BDCA3^+^ dermal DCs had up-regulated a damaged cell uptake receptor CLEC9A. No other infected epidermal cells interacted with dermal DCs. Correspondingly, LCs isolated from human skin and infected with HSV-1 *in vitro* also underwent apoptosis and were taken up by similarly isolated BDCA3^+^ dermal DCs and DC-SIGN^+^ cells. Thus, we conclude a viral antigen relay takes place where HSV infected LCs undergo apoptosis and are taken up by dermal DCs for subsequent antigen presentation. This provides a rationale for targeting these cells with mucosal or perhaps intradermal HSV immunization.

## Author Summary

Herpes Simplex Virus (HSV) is a highly prevalent virus that causes cold sores and genital herpes but also increases the chance of contracting HIV by several folds. In fact, most new cases of HIV in Africa occur in people infected with HSV. Thus, a protective HSV vaccine would have a large impact on public health. Currently, the process by which immunity to HSV is generated is incompletely understood. Paradoxically, the first immune cells to become infected, Langerhans cells in the epidermis, are not the cells that initiate the immune response, while the dermal dendritic cells thought to be responsible for initiating the immune response are not likely to be infected. Here, we have shown, in human skin models and genital herpes lesion biopsies, an interaction between these dendritic cells that could relay HSV to the lymph node. HSV is taken up by the epidermal Langerhans cells that then migrate into the dermis, die and are taken up by another subset of dermal dendritic cells—the homologs of those in mice which stimulate HSV-specific T cells in the lymph node. Thus, a mucosal or intradermal vaccine targeting these two dendritic cells may be required.

## Introduction

Dendritic cells (DCs) in the skin and mucosa play a major role as sentinels in the detection and uptake of pathogens and initiation of innate and adaptive immune responses [1]. Herpes Simplex Virus (HSV) types 1 and 2 are examples of closely related pathogens which invade the anogenital mucosa, penetrating into the stratified squamous epithelium especially where the overlaying stratum corneum is thin, absent or traumatically destroyed [2]. HSV-2 productively infects the epidermal keratinocytes [3, 4] and, as shown in mice, Langerhans cells (LCs) [5]. HSV-1/2 replication is restricted to the epidermis prior to infiltration of the virus into sensory nerve endings where it is transported to the dorsal root ganglion, resulting in lifelong latent infection of the neurons followed by intermittent reactivation leading to symptomatic lesions or asymptomatic shedding in the anogenital region [6]. Primary or initial genital herpes is defined as the first episode of herpetic lesions (HSV types 1 or 2) without pre-existing antibodies. Recurrent lesions of HSV can occur in the presence of high titres of neutralizing antibody [7, 8]. As recognized in patients with severe immunosuppression [9, 10], T cells play a major role in the control of both initial and recurrent herpes simplex [11].

In human recurrent herpes lesions, CD4 T cells infiltrate first, followed by CD8 T cells which correlates with termination of the infection [11, 12]. In mice, after clearance of initial infection, tissue resident memory CD8 T cells lodge in the epidermis close to the site of infection but CD4 memory T cells are found deeper in the dermis and remain migratory [13]. The human equivalent of the intra-epidermal tissue resident CD8 memory T cells lodges at the dermo-epidermal junction [14].

The sequence of events by which skin DCs take up HSV and present antigen to stimulate CD4 and CD8 T cells leading to development of memory T cells is more complex than first envisaged. In murine models, HSV is taken up by LCs in the epidermis [15] but they are not the antigen presenting cells to T cells in lymph nodes of mice. In mice, neither LCs nor resident DCs presented HSV-2 antigens to CD4^+^ T cells in lymph nodes but CD11c^+^ and CD11b^+^ submucosal DCs did [16]. Furthermore, naive CD8 T cells are primed by CD8α^+^ DCs and langerin^+^CD103^+^ dermal DCs [17–19] and the latter are the predominant cells transporting HSV antigens out of murine skin explants [5].

However, there have been no parallel studies in humans. Therefore, here we address whether viable HSV or HSV antigen is transferred from infected LCs in epidermis to the dermal DCs in three different human systems, explants of inner foreskin or of epidermis from abdominal skin, biopsies from initial human genital herpes lesions, and in LCs and dermal DCs isolated from skin.

The skin DC subsets have been well defined in mice. However, how human skin DCs correspond to mouse DC subsets is still being clarified. Recently, BDCA3^+^ (CD141^+^) DCs in human blood and skin have been recognized to be equivalent to murine CD103^+^/CD8α^+^ DCs [20, 21] in that both cell subsets express chemokine receptor XCR1 and damaged cell receptor CLEC9A as well as TLRs 1, 2, 3, 6, 7, 8, and possess high antigen cross-presentation capacities [22–24]. In human skin, they were much more efficient than other dermal DC subsets [17], although other DC subsets appear to be equally able to cross-present from other sites [25]. Presumably, the other non cross-presenting dermal DC subsets present exogenous HSV antigens to stimulate CD4 T cells as in mice [16].

Therefore, we first examined if LCs containing HSV or HSV antigen interact with underlying BDCA3^+^ dermal DCs in HSV-1 infected foreskin explants, upon topical infection of the inner surface which lacks a well-developed stratum corneum. For comparison, we also examined if such LCs interacted with (CD14^+^) dermal cells expressing DC-SIGN (CD209) especially as these have been reported to present antigen direct to CD4 T cells [26]. We chose DC-SIGN as a marker because the dermal DC subsets expressing BDCA3 and DC-SIGN appear to be mutually exclusive, unlike CD1a or CD1c which can be upregulated on BDCA3^+^ dermal DCs [21]. This strategy may miss some CD1c^+^ dermal DCs but most importantly, it provides a clear distinction between the BDCA3^+^ and another dermal DC subsets. More recently, these CD14^+^, DC-SIGN^+^ cells have been reported to more closely resemble dermal macrophages of monocyte origin although they are still potent antigen presenting cells [27]. The results were compared with similar skin LC-dermal DC interactions occurring within the biopsies of initial penile herpes simplex lesions, three days after onset of symptoms. The immunohistochemistry of primary herpetic lesions has not been previously studied. Finally, we examined the results of the in vitro interaction between LCs bearing HSV or HSV antigen with BDCA3^+^ dermal DCs, both isolated separately from skin, to determine whether these dermal DCs could take up LCs or cell fragments containing HSV or HSV antigen.

These results show a unique pathogen driven interaction, resulting in a pathogen/antigen relay from human LCs to two different subsets of antigen presenting dermal cells/DCs, occurring within induced clusters of cells in the dermis, with likely different roles in stimulating different effector T cell subsets, either in lymph nodes or later, infiltrating the dermis. This is the first report of a human study that shows dermal DCs may play a key role in delivering and presenting viral antigens to T cells in the lymph nodes as well as possibly to residential T cells.

## Materials and Methods

### Ethics statement

This study was approved by the Ethics Committee for Western Sydney Local Health District and written informed consent was received from participants or legal guardians of infants or young children aged under 14 prior to inclusion in the study.

### Virus preparation

v-UL37GFP (HSV-1 strain 17; gift from Dr. Frazer Rixon)[28] was prepared by infecting 80–90% confluent monolayers of African Green Monkey Kidney (Vero) cells. Infected cells were incubated in DMEM (Lonza) supplemented with 5% FBS (Sigma-Aldrich), harvested after 2 days and briefly sonicated using a cup horn sonicator (Branson) to release cell-associated virus. Virus was clarified by centrifugation at 20,000 x g for 10 min, supernatant was stored at -80°C until use. Virus titres were determined by plaque assay.

### Biopsies of initial human penile herpetic lesions

**Fig 1:**
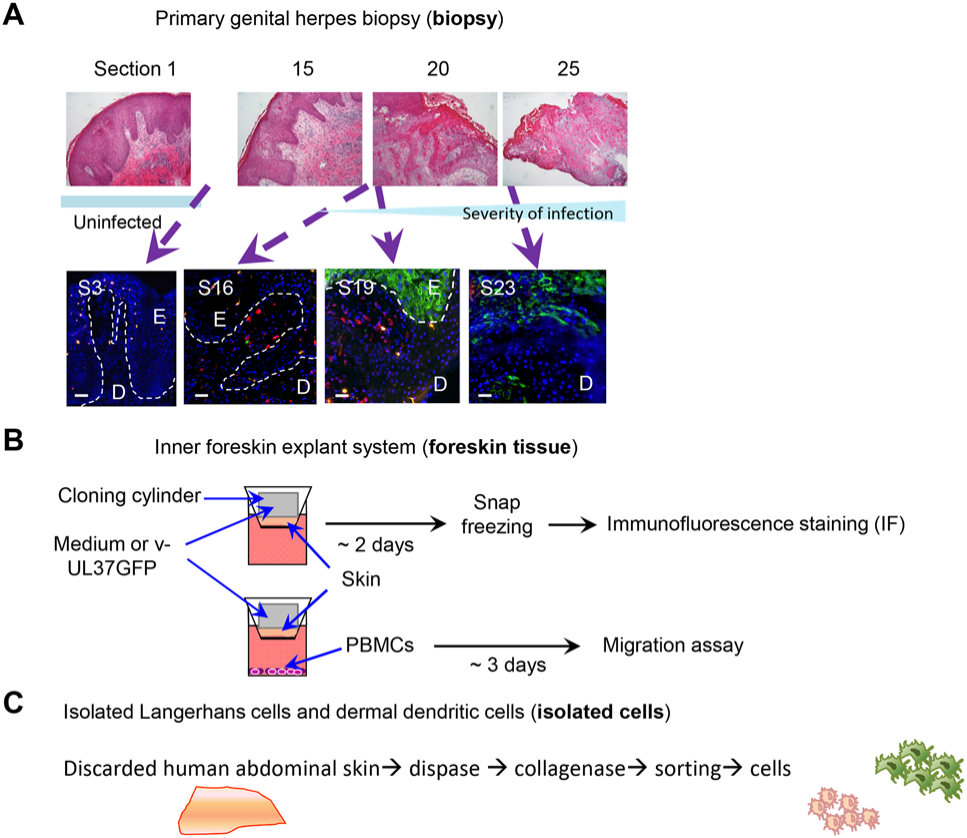
Schemes of experimental systems used to examine LCs and dermal DC interaction (A) Serial sections of a primary herpetic lesion stained by H & E (upper panels) and IF (lower panels). Blue: DAPI, green: HSV-1 glycoprotein D, orange: langerin, red: DC-SIGN. E: epidermis, D: dermis, S: section. Dotted line indicates basement membrane. Scale bar indicates 50 μm. (B) Scheme showing infection and processing the *ex vivo* inner foreskin tissue explant culture. (C) Scheme showing isolation of epidermal LCs and dermal DCs from abdominal skin.

3 mm punch biopsies of a primary and an initial non-primary penile herpetic lesions caused by HSV-1 and 2, respectively, were obtained at 3 days after onset. The edge of the biopsy passed through the centre of the lesion (Fig 1A). The biopsies were snap-frozen in OCT (ProSciTech) and kept at -80°C until cryosectioning. 7 μm cryosections were used for hematoxylin & eosin (H & E) and immunofluorescence (IF) staining.

### *Ex vivo* inner foreskin explants

Normal foreskin tissues were obtained from infants and young children undergoing circumcision. Tissues were wrapped in sterile gauze soaked with phosphate-buffered saline (PBS) and transported to the laboratory immediately following circumcision, and processed within the next 2 hr. Tissues were left in PBS containing 250 μg/ml gentamicin (Sigma-Aldrich) for 1 hr at room temperature then inner foreskin was separated from outer foreskin using a pair of scissors. Only inner foreskin tissues were used for this study as they lacked stratum corneum and did not require its mechanical removal for successful infection with HSV. Skin was cut into pieces >6 μm diameter and placed inside upper chambers of 24 transwell plates (5 μm pore size; Corning) with the epidermis facing up. Hollow plastic cloning ring cylinders of 6 mm inner diameter were topically applied to the epidermal side of each tissue piece, using vacuum silicone grease (Sigma-Aldrich) to contain HSV application to the superficial surface. To verify that the apical cloning ring cylinders were completely sealed, trypan blue solution (Gibco-BRL) was added to the inner space of the cylinders and incubated for 48 hr at 37°C. After incubation, trypan blue remained inside the cylinders without any leakage. RPMI 1640 (Lonza) supplemented with 10% human AB serum (Life Technologies) (RH10), 100 μg/ml streptomycin and 100 U/ml penicillin (Gibco-BRL) was added into the bottom chambers and plate was incubated for various times at 37°C (Fig 1B). Inner foreskin explants were exposed to 3 x 10^5^ v-UL37GFP or to RPMI 1640 as negative mock-infected control in the cloning ring cylinders and incubated for various times as indicated. After incubation, tissues were snap-frozen in O.C.T. (ProSciTech) and kept at -80°C until cryosectioning. Each block was cut into 7 μm sections and kept at -80°C until IF staining.

### Antibodies

Antibodies used for IF include: rabbit anti-GFP or HSV-1 glycoprotein D (gD; polyclonal; abcam), goat anti-langerin (monoclonal; R & D), mouse anti-BDCA3 or caspase3 (monoclonal; BD Biosciences), mouse anti-HSV-1/2 ICP27 or anti-DC-SIGN (monoclonal; abcam), mouse anti-CD80 (monoclonal; BD Pharmingen), rat anti-CLEC9A (monoclonal; gift from Dr. Mireille H. Lahoud); Alexa Fluor-488 conjugated donkey anti-rabbit IgG, Alexa Fluor-546 conjugated donkey anti-goat IgG, Alexa Fluor-647 conjugated donkey anti-mouse IgG (Molecular Probes).

Antibodies for flow cytometry include: AmCyan-anti-CD45, FITC-anti-CD1a, APC-anti-CD14, APC-anti-CD3, PE-Cy7-anti-HLA-DR, APC-anti-BDCA3, V450-anti-DC-SIGN (BD Biosciences), PE-anti-langerin (R & D).

### Immunofluorescence microscopy

For in vitro experiments, co-cultured LCs and dermal DCs as above were stained for IF as previously described [29] except that primary antibodies were diluted in Protein Block Serum Free buffer (DAKO) and PBS/0.05% Tween 20 was used as the first wash for each washing step. For inner foreskin and primary genital herpetic lesion biopsy tissues, slides were fixed and permeabilized with ice-cold methanol:acetone (1:1) for 10 min prior to blocking with 10% normal donkey serum for 20 min at room temperature.

Cells with primary and secondary antibodies were incubated at 37°C for 45 min and 30 min, respectively. ProLong Gold reagent with DAPI (Invitrogen) was used for nuclear staining. Images were acquired with an inverted Olympus IX-70 microscope (DeltaVision Image Restoration Microscope; Applied Precision/Olympus) and a Photometrics CoolSnap QE camera, and analyzed using FIJI and DeltaVision SoftWorx deconvolution software 5.5.1 RC3.

### Flow cytometry

Flow cytometry was performed on a BD LSR Fortessa and cell sorting using a BD Influx. For cell phenotyping, at least 15,000 events were acquired. Data were analysed using FlowJo (TreeStar). Antibodies for flow cytometry and cell sorting are detailed below with individual experiments.

### Isolation of LCs and dermal DCs from human skin and their co-culture with or without HSV infection of LCs

Discarded skins were obtained from surgical apronectomy and processed as previously described (Fig 1C) [30]. Epidermal cells were labelled with DAPI (Invitrogen), CD45, CD1a, CD14, CD3 antibodies for flow sorting LCs. DAPI^-^CD14^-^CD3^-^ cells were gated to sort CD45^+^CD1a^+^ LCs. Dermal skin pieces were washed with MACs washing buffer containing 20 μg/ml DNase and filtered before sorting for live HLA-DR^+^BDCA3^+^ dermal DCs.

Sorted LCs were pulsed with v-UL37GFP (MOI 10) or RPMI 1640 media for 2 hr and then washed with RPMI 1640 before incubating for 12 hr in RH10 supplemented with 200 ng/ml GM-CSF (Invitrogen) and 25 μg/ml gentamicin. After incubation, LCs were washed in RPMI 1640 and then co-cultured with sorted BDCA3^+^ dermal DCs for 6 hr. Sorted BDCA3^+^ dermal DCs were incubated in the same media until co-culturing with HSV-1 infected LCs.

### Cell migration assay

For the cell migration assay, inner foreskin explant tissues were incubated for 72 hr as above, with or without allogeneic PBMCs placed in the bottom chambers in RH10 (Fig 1B). After 72 hr, cells were collected from bottom wells and labelled with CD45, HLA-DR, langerin, BDCA3, DC-SIGN antibodies. Propodium iodide (PI; 1 μg/ml; BD Biosicences) was added just before acquiring cell events.

### Statistical analysis

**Fig 2:**
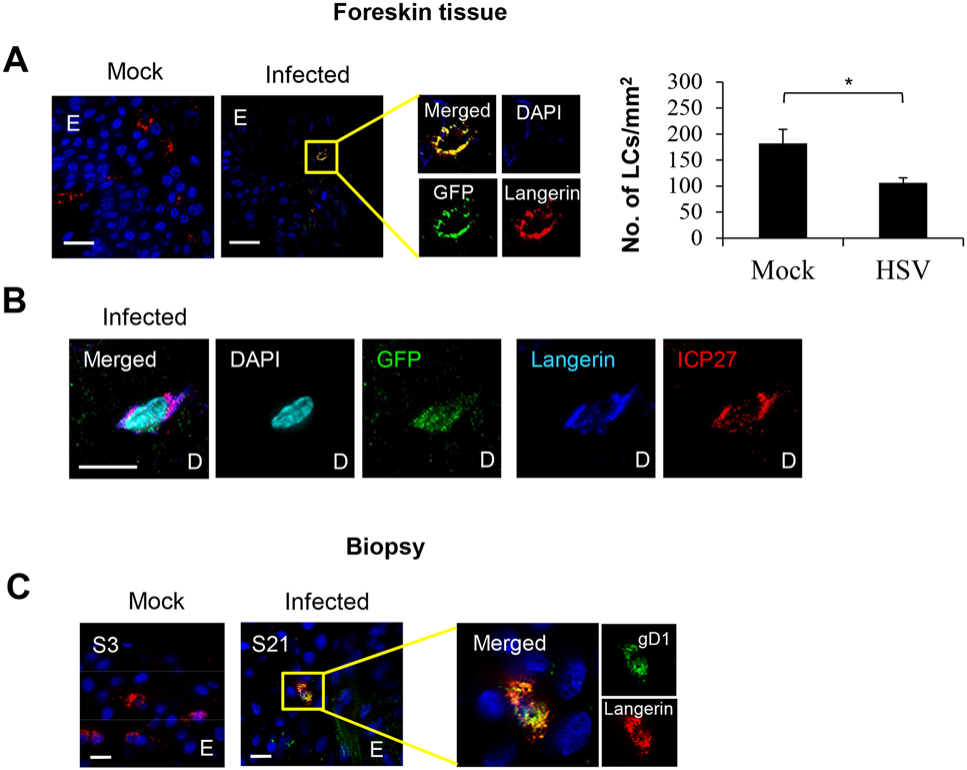
HSV-1 infected LCs in human skin. (A) LCs in the epidermis of mock or HSV-1 infected inner foreskin explants. The right panel quantifies density of total LCs in the epidermis of mock and HSV-1 infected explants at 24 hr p.i. in 20 representative fields per sample at 60x magnification. n = 3, mean ± SEM, *p<0.05. (B) ICP27 expression of the HSV-1 infected LC emigrated into the dermis of the inner foreskin explant. ICP27: immediate early protein of HSV, D: dermis. Representative result of 3 different donors is shown. (C) LCs in the primary penile herpetic lesion. E: epidermis, gD1: HSV-1 glycoprotein D. Representative result of 2 different donors. Scale bar indicates 20 μm. Maximum projections of Z-series are presented.

**Fig 3:**
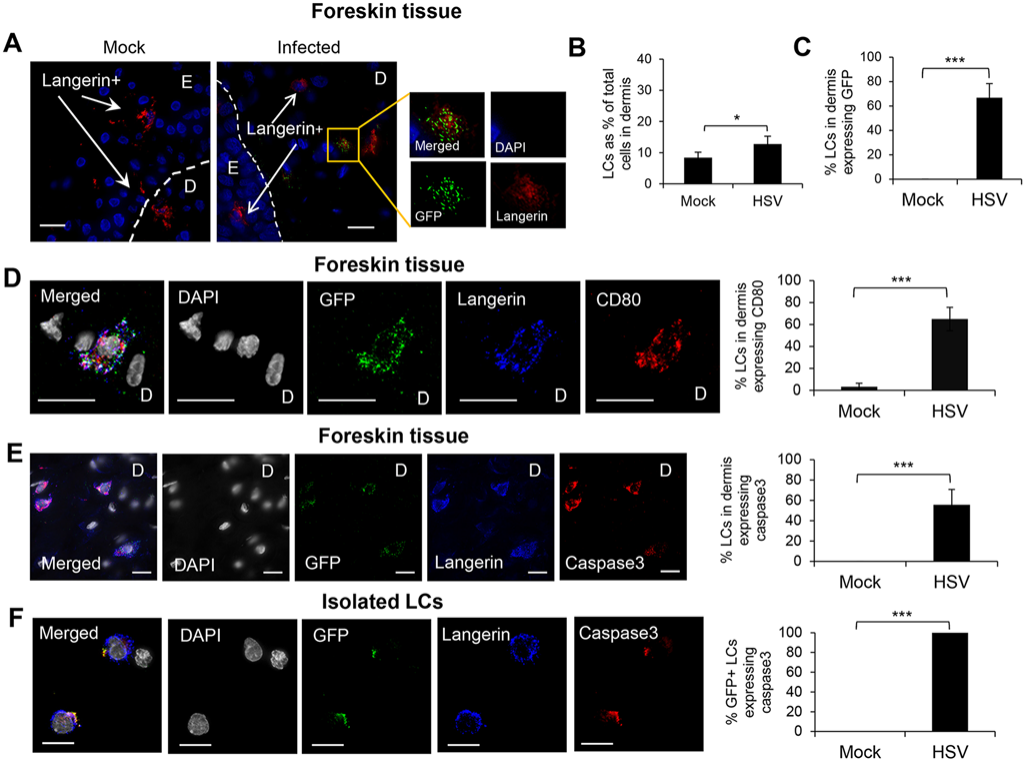
Fate of human LCs infected with HSV-1. Inner foreskin explants (A, B, C, D & E) or isolated skin LCs (F) were infected with v-UL37GFP for 24 hr (A, D, & E), 48 hr (B & C), or 18 hr (F). (A) LCs in the dermis of inner foreskin explants. Dotted line represents basement membrane. (B) Comparison of LCs migrating into the dermis in mock and infected inner foreskin explants expressed as % LCs per total no. of dermal cells. n = 6, mean ± SEM, *p<0.05. (C) Proportion of LCs in the dermis of inner foreskin explants expressing GFP, n = 3, mean ± SEM, ***p<0.001. (B) (C) LCs with or without GFP expression were quantified in 20 representative fields per sample at 60x magnification. (D) (E) Infected LCs in the dermis of inner foreskin explants were tested for the expression of a maturation marker CD80 (D) or an apoptosis marker caspase 3 (E). (F) Infected LCs isolated from abdominal skin were examined for the expression of caspase 3. Right-hand panels show quantification of each marker for (D), (E), (F) as in (B) and (C), n = 3, mean ± SEM, ***p<0.001. Maximum projections of Z-series are presented (D, E & F). E: epidermis, D: dermis. Scale bar indicates 15 μm.

**Fig 4:**
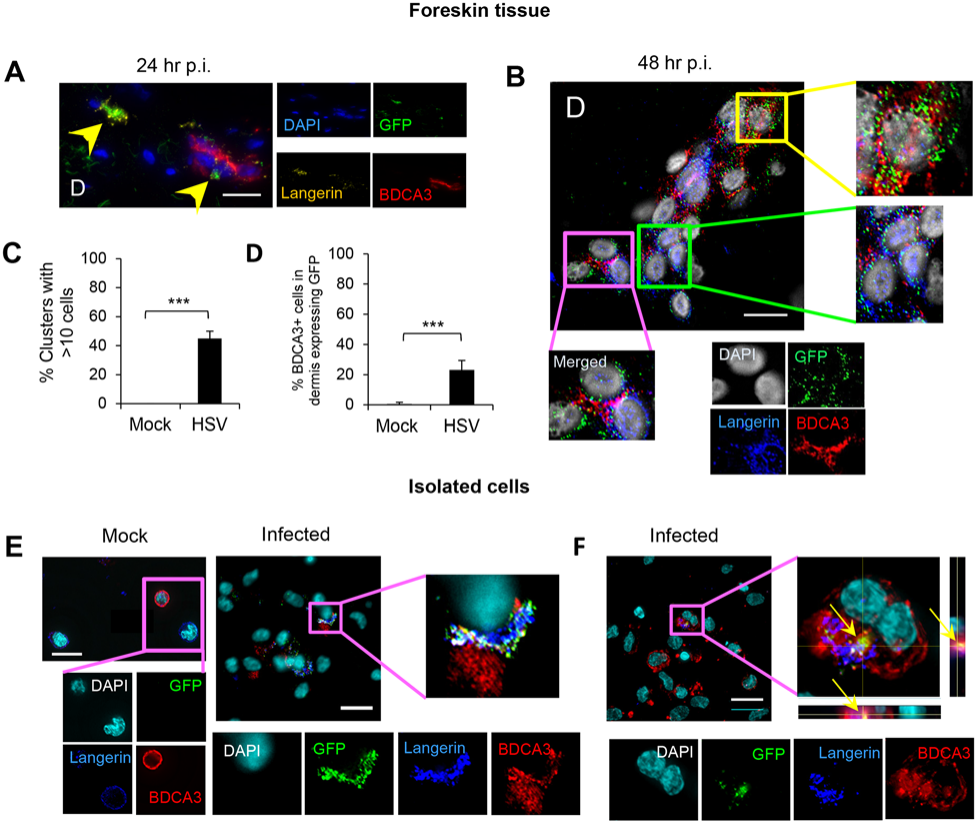
Migration of HSV infected LCs into the dermis and their subsequent interaction with BDCA3^+^ dermal DCs in clusters. (A) (B) (C) (D) LCs and BDCA3+ dDCs in the dermis of foreskin explants at 24 hr p.i. (A) & 48 hr p.i. (B, C, & D), yellow arrows: GFP^+^langerin^+^ cells, green box: GFP^+^langerin^+^ cells, yellow box: GFP^+^BDCA3^+^ cells, pink box: GFP/langerin^+^BDCA3^+^ dermal DCs. (C) Proportion of GFP^+^LC/BDCA3^+^ DC clusters with >10 cells, n = 3, mean ± SEM, p***<0.001. (D) Proportion of BDCA3^+^ dermal DCs expressing GFP, n = 3, mean ± SEM, p***<0.001. BDCA3^+^ cells with or without GFP expression were quantified in 20 representative fields per sample at 60x magnification. (E) (F) Isolated skin LCs and BDCA3^+^ dermal DCs: LCs were pulsed with v-UL37GFP for 2 hr, incubated for 12 hr then washed before co-culturing with BDCA3^+^ dermal DCs for another 6 hr. Images were acquired by maximal intensity Z projection (A, B, E & F) and orthogonal views with the xz and yz planes (F). Scale bar indicates 20 μm. Images are representative from 3 donors.

**Fig 5:**
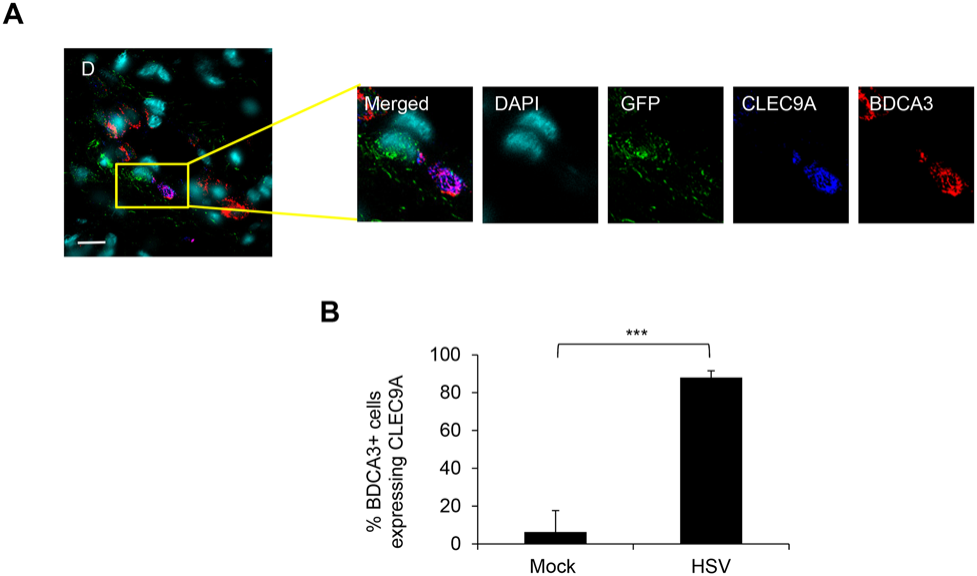
CLEC9A expression by BDCA3^+^ dermal DCs in HSV-1 infected foreskin explants. Inner foreskin explants were infected with or without v-UL37GFP for 48 hr. (A) Representative image from 3 different donors is shown. D: dermis. Scale bar indicates 15 μm. (B) CLEC9A^+^BDCA3^+^ cells were quantified in 20 representative fields per sample at 60x magnification from 3 donors, mean ± SEM, p***<0.001.

**Fig 6:**
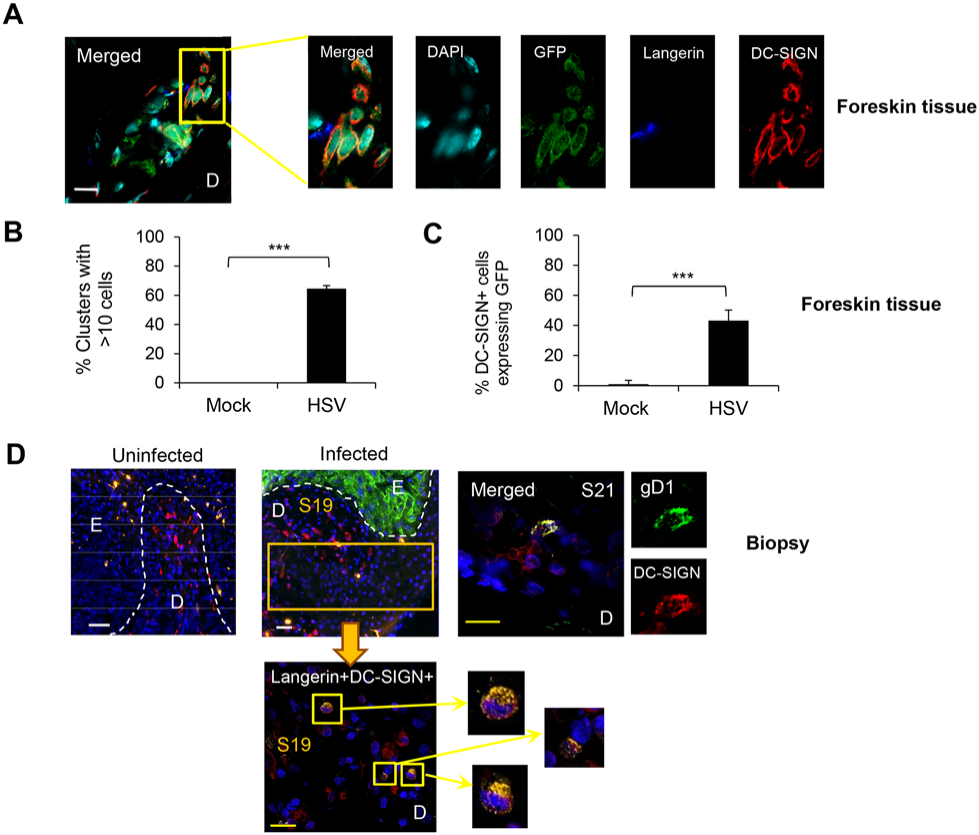
Interaction of HSV-1 infected LCs with DC-SIGN^+^ dermal cells (dermal DCs/macrophages). (A) (B) (C) Foreskin explants, 48 hr p.i. (A) LCs and DC-SIGN^+^ dermal cells interacted in clusters. (B) Proportion of clusters GFP^+^LC/DC-SIGN^+^ dermal cells containing >10 cells, n = 3, mean ± SEM, p***>0.001. (C) DC-SIGN^+^ dermal cells with or without GFP expression were quantified in 20 representative fields per sample at 60x magnification from 3 separate samples. Mean ± SEM, p***>0.001 (D) Primary penile herpetic lesion, blue: DAPI, orange: langerin, red: DC-SIGN, green: gD1. E: epidermis, D: dermis. The dotted line represents the basement membrane. Maximum projections of Z-series are presented (A & D). Scale bar in (A) indicates 15 μm and scale bars in (D) indicate 50 μm (white) and 15 μm (yellow), respectively.

**Fig 7:**
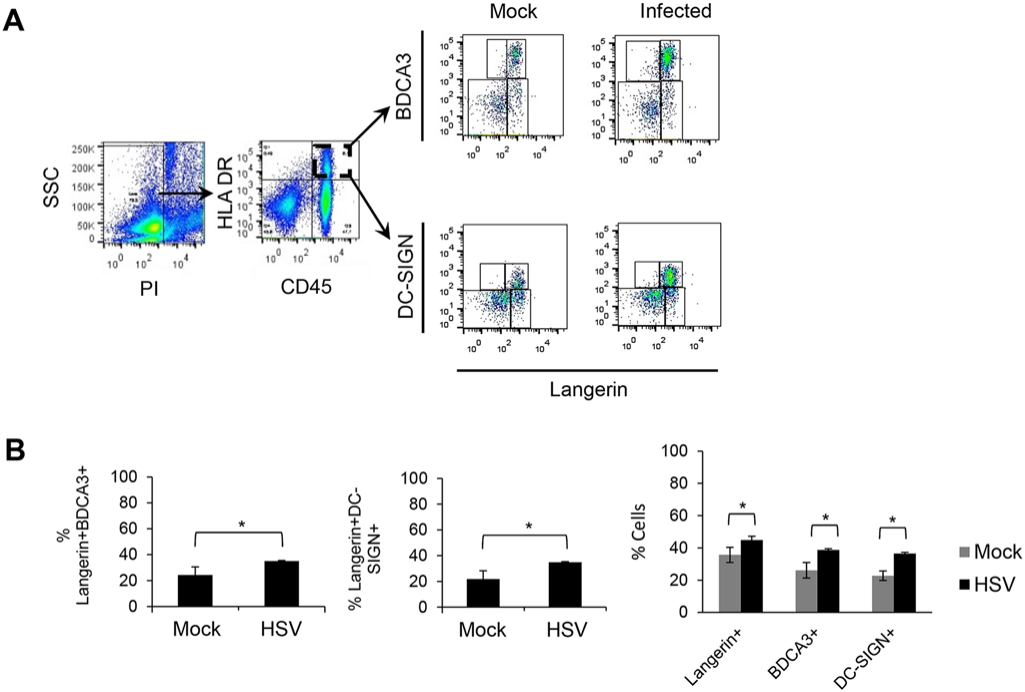
Emigration of DCs from inner foreskin explants with or without HSV infection. Inner foreskin explants were cultured for 72 hr in 24 transwell plates (membrane with 5 μm diameter pore-size) with or without v-UL37GFP. Tissues were placed in the upper chamber and allogeneic PBMCs were placed in the bottom chamber as shown in Fig 1B. After 72 hr, cells were collected from bottom chambers and labelled for flow cytometry. (A) Cells were gated on viable cells (PI^-^) then CD45^+^HLA-DR^+^ cells before identifying different subsets of DCs migrated out of tissues to the bottom chambers. (B) The percentage of viable CD45^+^HLA-DR^+^ cells co-expressing langerin and BDCA3 or DC-SIGN and the percentage of individual emigrated cells in the bottom wells of mock or infected samples are shown. Repeated measures ANOVA was used to test statistical significance, n = 3, mean ± SEM, p*<0.05.

For pairwise comparisons between mock and infected between foreskin samples in Figs 2, 3, 4, 5 and 6, statistical analyses were done by two tailed paired student *t* test, adjusted for unequal variances. For Fig 7B, repeated measures ANOVA was used to assess the within DC subset effects of treatment and cell type. SPSS version 21 was used to analyze data. 5% significance level with two-sided tests was used throughout.

## Results

### System for examining LC infection by HSV and their subsequent interactions with dermal DCs in skin

HSV uptake and infection of LCs were examined in three main experimental systems, inner foreskin explants, in two biopsies of initial herpetic lesions (one primary), in immature LCs and dermal DCs isolated from abdominal skin (Fig 1). The interaction of LCs with dermal DCs was then examined in all systems. In addition, HSV infection of isolated epidermal sheets from abdominal skin allowed examination of emigrating LCs with or without HSV infection (S1 Fig).

### Patients and biopsies

The first biopsy was taken from a 31 year old male who presented with a 3 day history of a lesion on the penile shaft, originally papular then ulcerating. He had no prior history of facial or genital herpes. HSV-1 was isolated from the lesion. Serology showed an absence of HSV IgM and IgG at the time when the 3 mm diameter punch biopsy was taken overlapping the center of the lesion. H & E stained sections at intervals (sections 1, 15, 20, 25 of 25) showed a transition from the uninflamed part (IF section 3) to the fully developed ulcerative lesion (IF section 23). In section 20 stained by H & E, dermal mononuclear cell infiltrates are shown close to the center of the lesion and in section 25, epithelial ulceration and severe necrosis are obvious with partial loss of basement membrane. In IF section 19, the epidermis was still intact (despite being close to the center of the lesion) but IF showed HSV gD expression by epidermal cells throughout its full thickness (Fig 1A).

The second biopsy was taken from a 44 year old male who presented with a 3 day history of a lesion on the penile shaft. He had no prior history of facial or genital herpes. PCR for HSV-2 DNA was positive. Serology showed an absence of HSV IgM for HSV-1 and 2 but there was pre-existing IgG to HSV-1 at the time of biopsy.

### HSV-1 infected human LCs in the epidermis of infected inner foreskin explants and initial herpes simplex lesions

To examine whether HSV can infect human LCs, we used GFP-labelled HSV-1, v-UL37GFP, to infect inner foreskin explants in situ and compared it with initial penile herpetic lesion biopsies of the HSV-1/2 infected males at 3 days post onset (Fig 2). In the epidermis of human inner foreskin explants at 24 hr after topical application of v-UL37GFP, GFP was detected in subjacent LCs remaining in the epidermis (Fig 2A), quantified in 20 fields to be 38% of LCs. However, the density of LCs remaining in the epidermis was significantly lower in HSV infected than mock infected explants (Fig 2A right panel), complementing the increased density of migrating LCs found in the dermis (Fig 3B). The proportion of infected LCs is quite similar to that quantified by flow cytometry in LCs emigrating onto substrate after infection of the explant (28%) with monoclonal antibody targeting HSV glycoprotein C (gC) (S1 Fig). To determine whether there was viral replication as well as uptake of HSV by LCs in infected foreskin explants, we examined the expression of ICP27 which is an immediate early non-structural viral protein and therefore represents HSV replication [31]. ICP27 was also expressed in LCs which emigrated into the dermis of inner foreskin explants 24 hr post infection (p.i.) (Fig 2B) but was rarely detected in any other surrounding dermal DCs. Similar to the results with foreskin explants, the HSV-1 late protein, glycoprotein D (gD1) was expressed in LCs remaining in the epidermis of the initial herpetic lesions (Fig 2C) and also in infected epidermal keratinocytes shown in Fig 1A and Fig 6D.

### GFP^+^ LCs migrated into the dermis, where they expressed CD80 and/or underwent apoptosis

In foreskin explants infected with v-UL37GFP, GFP^+^LCs were found in the dermis, most in the upper dermis, within 24 hr p.i. (Fig 3A). At 48 hr p.i., uninfected LCs were also observed to migrate into the dermis but at a significantly lower frequency (Fig 3B). At 48 hr p.i., 67% LCs which migrated into the dermis of infected explants expressed GFP (Fig 3C).

The maturation status of migrated GFP^+^ LCs in the dermis was examined. Greater than 60% of GFP^+^LCs, and only they, were found to express the costimulatory molecule CD80, associated with a mature phenotype, at 24 hr p.i. (Fig 3D). GFP^+^ LCs in the dermis often exhibited a shrunken and occasionally fragmented morphology suggesting they were apoptotic, so we examined whether such HSV infected LCs expressed caspase 3 as a marker for apoptosis. Greater than 55% of GFP^+^ LCs expressed caspase 3 in the dermis of the infected inner foreskin explants but none in the mock infected explant (Fig 3E). When LCs were isolated from the epidermis of abdominal skin and infected with v-UL37GFP in vitro, all infected but no mock infected cells expressed caspase 3 at 18 hr p.i. (Fig 3F).

### GFP^+^ LCs interacted with BDCA3^+^ dermal DCs in the dermis

GFP^+^ LCs and BDCA3^+^ dermal DCs were observed to interact in clusters in the dermis of infected inner foreskin explants. At 24 hr p.i., most GFP^+^ LCs maintained intact morphology, sometimes adjacent to BDCA3^+^ dermal DC clusters (Fig 4A). However, at 48 hr p.i., GFP^+^langerin^+^ cells/cellular fragments were intermingled with BDCA3^+^ dermal DCs in large clusters (Fig 4B). In those clusters, various types of co-localization were observed which were GFP^+^BDCA3^+^, GFP^+^langerin^+^, or GFP^+^langerin^+^BDCA3^+^ cells (yellow, green, and pink boxes in Fig 4B, respectively). At 48 hr p.i., greater than 45% of the GFP^+^LC/BDCA3^+^ DC clusters consisted of greater than 10 cells (Fig 4C). In mock infected explants, no LC/BDCA3^+^ DC clusters were observed although small clusters (<10 cells) of BDCA3^+^ cells alone were infrequently observed. At 48 hr p.i., more than 20% of BDCA3^+^ DCs in infected explants expressed GFP (Fig 4D). There was no ICP27 staining or GFP expression in BDCA3^-^ cells surrounding these clusters suggesting that viral replication in LCs was contained, possibly by apoptosis, and had not spread to surrounding fibroblasts.

### Interaction of HSV infected LCs isolated from skin with BDCA3^+^ dermal DCs *in vitro*

As we observed clustering of GFP^+^ LCs and BDCA3^+^ dermal DCs in the foreskin dermis, and BDCA3^+^ dermal DCs are known to be specialized for uptake of apoptotic/necrotic cells or fragments and cross-presention to CD8 T cells, we decided to examine this further in vitro. LCs and dermal DCs were isolated from large abdominal skin specimens by collagenase digestion then sorted (S2 Fig). When LCs were pulsed with v-UL37GFP for 2 hr then incubated for 12 hr and co-cultured with BDCA3^+^ dermal DCs for 6 hr, co-localization of GFP, langerin and BDCA3 were observed in infected samples but not in mock infected samples (Fig 4E). Approximately 50% of BDCA3^+^ cells were co-localized with GFP^+^langerin^+^ cells. Rarely, GFP^+^langerin^+^ cell fragments were found inside BDCA3^+^ DCs (Fig 4F). As only LCs were exposed to v-UL37GFP, these observations verified uptake of infected LCs by BDCA3^+^ dermal DCs.

### CLEC9A expression by BDCA3^+^ dermal DCs was more frequent in HSV-1 than mock infected inner foreskin explants

As CLEC9A is known as a damaged cell recognizing receptor on human blood BDCA3^+^ dermal DCs, we examined CLEC9A expression in BDCA3^+^ dermal DCs in HSV-1 infected inner foreskin explants. In HSV-1 infected foreskins explants, CLEC9A was expressed in BDCA3^+^ dermal DCs adjacent to GFP^+^ cells which were almost certainly LCs (Fig 5A). This could not be confirmed because of limitations on the number of simultaneous fluorescent antibodies. However, only LCs and dermal DCs were observed to express GFP in the dermis of HSV-1 infected inner foreskin explant tissues throughout our study. Although blood BDCA3^+^ DCs constitutively express CLEC9A, this was not the case with dermal BDCA3^+^DCs. There was a marked difference in the proportion of BDCA3^+^ DCs expressing CLEC9A between mock (6%) and infected (88%) inner foreskin explants (Fig 5B). This observation indicated that there were damaged cells, most probably LCs in the dermis of HSV infected inner foreskin tissues adjacent to BDCA3^+^ dermal DCs.

### GFP^+^ LCs interacted with DC-SIGN^+^ dermal cells

We also examined the interaction of GFP^+^ LCs with DC-SIGN^+^ dermal DCs/macrophages to complement BDCA3, as DC-SIGN is expressed on a distinctly separate dermal DC/macrophage subset by IF (S3 Fig). Approximately 40% of DC-SIGN^+^ cells expressed either GFP or langerin or both in the dermis of HSV-1 infected foreskin explants at 48 hr p.i. (Fig 6A and 6C). In infected explants, similar to BDCA3^+^ dermal DCs, large GFP^+^LC/DC-SIGN^+^ cell clusters formed: greater than 60% of these cell clusters consisted of >10 cells whereas none were observed in mock infected explants (Fig 6B). Again, small clusters of DC-SIGN^+^ DCs alone were observed. Similarly, there was no GFP expression in langerin^-^ or DC-SIGN^-^ cells surrounding the clusters, indicating no spread to surrounding fibroblasts. In the dermis of the initial penile herpetic lesions, HSV gD and/or langerin expressing DC-SIGN^+^ cells were also observed (from section 19, Fig 1) but widespread. HSV-1 infection was restricted to the keratinocytes of the epidermis (Fig 6D).

### Migration of DCs out of inner foreskin explant tissues

As we observed emigration of GFP^+^ LCs from the epidermis towards BDCA3^+^ dermal DCs or DC-SIGN^+^ dermal DCs/macrophages in the dermis of infected inner foreskin explants, we next examined which cells actually migrated out of the dermis with or without infection using a transwell system. The transwell plate had pores of 5 μm diameter in the membrane between the upper and lower chambers to allow cell migration through the membrane. Allogeneic PBMCs were placed in the lower chamber as they were found to increase viability of emigrating DCs (S4 Fig) and to examine the interaction of emigrated DCs with PBMCs. There was little survival/emigration of DCs without PBMCs after 72 hr (S4 Fig) but significant emigration of LCs, BDCA3^+^ dermal DCs and DC-SIGN^+^ dermal DCs/macrophages from inner foreskin explants was observed when PBMCs were present in the lower wells, and to a greater degree in the infected samples (Fig 7A). Besides CD45^+^ leukocytes, a substantial proportion of the emigrating cells were CD45^-^, almost certainly keratinocytes [32, 33]. Among the CD45^+^HLA-DR^+^ cells in the lower chamber, 26% of emigrated BDCA3^+^ dermal DCs and 23% DC-SIGN^+^ dermal cells also expressed langerin markers, in significantly greater numbers (37% for emigrated BDCA3^+^ and 36% for emigrated DC-SIGN^+^) in infected samples than mock infected samples (Fig 7B). In PBMC only controls, no highly expressing DC-SIGN^+^ cell or BDCA3^+^ cells were observed at 72 hr p.i. so there was no confounding persisting population from the PBMCs.

## Discussion

Our studies show that after topical infection of human genital skin, there is a relay of HSV or HSV antigen between epidermal and dermal DCs. They also define the nature and site of the interaction of these DCs. Firstly, these human LCs were able to be infected in situ in the epidermis by HSV-1, in vitro as isolated LCs, *ex vivo* in infected foreskin explants and in vivo in a primary and an initial herpes biopsies, using expression of both structural (UL37, gD, and gC) and non-structural (ICP27) viral proteins. The expression of ICP27 implies that LCs not only take up HSV antigens but also become infected. We quantified the proportion of LCs expressing HSV proteins as a tegument protein UL37 in the epidermis at 24 hr p.i. to be 38% and as gC emigrating from isolated epidermal sheets from abdominal skin onto substrate by flow cytometry at 48 hr p.i. as 28%.

Then, in the infected inner foreskin explants, HSV infected or antigen bearing LCs were observed in the upper dermis by 24 hr p.i and eventually emigrated through the dermis to media within 72 hr after infection. Furthermore, LCs emigrating into the dermis of infected foreskin explants at 24–48 hr p.i., also expressed ICP27, indicating that HSV was actually replicating in the infected LCs. Uninfected HSV antigen negative LCs migrated less frequently into the dermis of inner foreskin explants and eventually out of skin at 48–72 hr p.i. The kinetics of such LC emigration is consistent with previous reports in HSV-1/2 infected murine models [5, 34]. These observations suggest that keratinocytes and LCs (which comprise only less than 1% of epidermal cells) are infected at the same time (within one replication cycle) without needing lateral spread from keratinocytes to LCs, although this may occur later. No infected cells other than LCs were observed to migrate into the dermis and interact with dermal DCs. The fate of infected LCs seems to be similar in situ in the explants and in LCs isolated from epidermal sheets of human abdominal skin by collagenase digestion in that they both show evidence of apoptosis. This is consistent with our previous observations of the effect of HSV infection of MDDCs in vitro [35] and in LCs in mice [5]. Although infected LCs were present in the dermis, there was no evidence of infection of surrounding dermal fibroblasts, suggesting that there was restriction of HSV spread, probably by apoptosis [36].

Furthermore, LCs expressing HSV-1 antigen also expressed CD80 and thus matured while migrating into the dermis. This differs from our previous work showing down-regulation of maturation markers after HSV infection of immature MDDCs [35]. Such discrepancies reflect different influences on LCs successfully emigrating into the dermis which overcome the effects of HSV infection per se on maturation, probably via cytokines released from infected keratinocytes.

The key and unexpected finding of this study was the nature of interaction of HSV infected and/or antigen expressing LCs with BDCA3^+^ dermal DCs and also DC-SIGN^+^ dermal cells. This sometimes involved the formation of clusters of as many as 10 dermal DCs of either subset closely interacting with HSV^+^ LCs or fragments of them in the dermis. HSV expressing LCs were observed to interact with such dermal DC clusters at 48 hr p.i.

In the dermis, BDCA3^+^ DCs are more efficient than other types of DCs (or macrophages) for cross-presentation [21]. CLEC9A is a novel activation C-type lectin-like receptor which is constitutively expressed on blood BDCA3^+^ DCs [37, 38] and known to recognize actin filaments on damaged/apoptotic cells [37, 39]. Unlike blood BDCA3^+^ DCs, our results showed that CLEC9A was not constitutively expressed on BDCA3^+^ dermal DCs but, in these clusters, appeared to be induced by adjacent GFP^+^ LCs which were also expressing apoptosis marker caspase 3. Thus, skin BDCA3^+^ dermal DCs differ in this respect to blood BDCA3^+^ DCs. Moreover, GFP labelled HSV pUL37 was also detected within BDCA3^+^ DCs and co-localized with langerin suggesting uptake of infected LC fragments. At 48 hr p.i., HSV infected LCs lost morphology and were fragmented. To confirm this observation, we used LCs and dermal DCs isolated from skin for sequential infection of LCs and co-culture with dermal DCs. Collagenase treatment was used to isolate immature epidermal and dermal DCs to avoid emigration associated maturation and reduced phagocytosis. The uptake of HSV infected LCs by BDCA3^+^ dermal DCs was confirmed by co-culturing HSV infected LCs and uninfected BDCA3^+^ dermal DCs isolated from abdominal skin. This is the first report of uptake of virus infected LCs undergoing apoptosis by human BDCA3^+^ dermal DCs. Further investigations to define the mechanism are needed in future.

BDCA3^+^ dermal DCs were observed to emigrate from HSV-1 infected explants, similar to CD103^+^ DCs in murine models [5]. They were detected in the lower chambers of transwells after culture for 72 hr, more frequent in infected samples, but only where allogeneic PBMC were present in the lower chamber. This suggests that such emigration of dermal DCs or viability after emigration is facilitated by cytokines/chemokines secreted by the PBMCs.

DC-SIGN is expressed exclusively on CD14^+^ dermal cells, not on CD1a^+^ or BDCA3^+^ dermal DCs [40]. DC-SIGN^+^ dermal cells interacted with GFP^+^ LCs somewhat similarly to BDCA3^+^ dermal DCs in all systems, in skin explants, the biopsy and in vitro isolated DCs. However, the pattern of co-localization of DC-SIGN, GFP and langerin was different to those in BDCA3^+^ dermal DC/GFP^+^ LC clusters in that langerin molecules were often absent in GFP^+^ LC/DC-SIGN^+^ cells clusters. This suggested that LC fragments and langerin are degraded more rapidly than HSV antigens inside the dermal DCs. Like BDCA3^+^ dermal DCs, DC-SIGN^+^ dermal cells, with or without langerin expression, also emigrated out of infected foreskin explants into the lower chamber.

Thus, these studies show that LCs take up HSV, become infected and pass live virus or antigen onto dermal DCs through cell clusters in the dermis. It provides an explanation for CD103^+^ DCs in mice carrying most HSV antigens out of skin and as the principal antigen presenting cells in draining lymph node [5]. BDCA3^+^ dermal DCs may present HSV antigens predominantly to CD8 T cells in the lymph nodes. The exact role of DC-SIGN^+^ DCs/macrophages after HSV uptake in initial HSV infection requires further study as they were recently shown to be weak at primary antigen presentation to naive T cells but strong stimulators of memory T cells [27].

**Fig 8:**
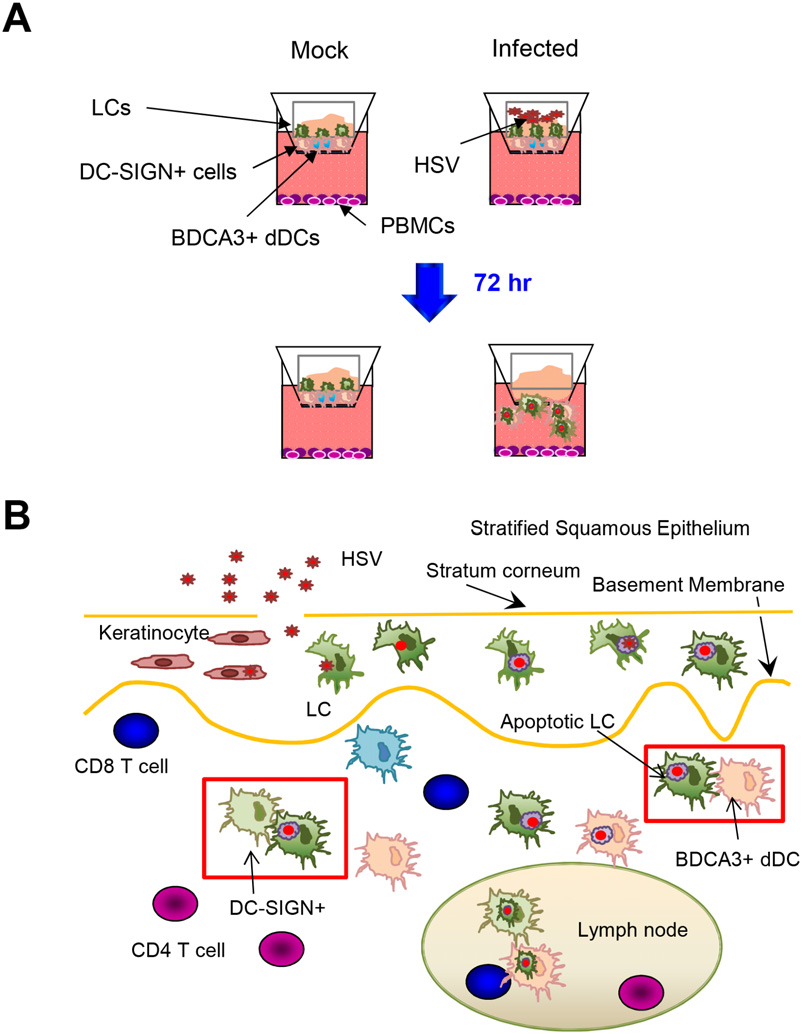
Migration and/or interaction of HSV infected human LCs with dermal DCs. (A) Migration of LCs and dermal DCs out of skin after topical HSV application to inner foreskin explants. (B) Summary diagram of interaction between HSV infected LCs and dermal DCs in human skin.

Our findings and hypothesis are summarised in Fig 8. Our study provides valuable insights into the immunopathogenesis of HSV primary infection in human skin and mucosal tissues. It also probably applies to recurrent herpes lesions where HSV is shed from nerve terminals in the mid-epidermis (stratum granulosum) in the vicinity of LCs which may become infected and migrate in a similar fashion. This work also provides a more general paradigm for uptake of cutaneous viruses or attenuated viral vaccines into epidermal LCs and subsequent interactions with dermal DCs prior to antigen presentation to naive or memory CD4 and CD8 T cells in the lymph node or resident in the skin or mucosa.

Furthermore, many unanswered questions from this study open up new fields of investigation. Firstly, there are few published data on cutaneous DC cross-talk. What are the mechanisms of cluster formation between LCs and dermal DCs, and what is the loss of the selectivity in LC-BDCA3^+^ DC vs LC-DC-SIGN^+^ cell interaction? Furthermore, how is LC/dermal DC maturation coupled to migration throughout the matrix of HSV infected skin. The reported dichotomy of BDCA3^+^ dermal DC emigration but not of dermal CD14^+^ cells into lymphatic vessels could be examined in a natural infection setting in our inner foreskin explant system [27]. What is the rate of virus/protein antigen degradation in migrating LCs and dermal DCs? HSV protein degradation appears to be slow in LCs. Finally, is a LC-dermal DC exchange a general response to epidermal viral or other pathogens? How does this exchange occur when viruses also invade the dermis as well as the epidermis, as with Varicella Zoster viruses?

Taken together, we conclude that LCs are not the carriers of HSV antigens to the lymph nodes but are involved in an ‘antigen relay’ after uptake by BDCA3^+^ and other dermal DCs or macrophages. BDCA3^+^ or DC-SIGN^+^ dermal DCs taking up HSV antigen via LCs may be able to present HSV antigen to T cells in the lymph nodes and also the dermis itself after CD4 and CD8 T cell infiltration. Moreover, the inner foreskin model is useful to study initial interactions of epithelia tropic viruses with human skin. Our study provides further insights into the immunopathogenesis of HSV primary infection in *ex vivo* human skin tissues and provides a useful tool to study the dynamic interactions of different cells encountering viruses in human skin. It also suggests that an intradermal or mucosal vaccine would be effective against HSV infection or disease particularly targeting BDCA3^+^ dermal DCs to enhance CD8 T cell responses, needed to complement the current vaccine candidates. Intradermal targeting of dermal DCs by subunit vaccines (e.g. single recombinant protein) may require simulation of the immune effects of HSV infected apoptotic LCs by adjuvants.

## Supporting Information

S1 Fig HSV infection of LCs in human discarded abdominal skin. Epidermal sheets obtained from apronectomy were infected with HSV-1 (strain F) at an MOI of 5 for 2 hours at 37°C to allow virus penetration. After the incubation period, epidermal sheets were washed three times in PBS and then cultured for 24–48 hours in RPMI 1640 supplemented with 10% FBS and 25 μg/ml of gentamicin. Cells crawling out from the epiderminal sheets were collected and stained with monoclonal antibodies against langerin (R & D), HLA-DR (BD Biosciences) and HSV-1 glycoprotein C (gC; ViroStat). 28% of the emigrating LCs expressed gC at 48 hr p.i. Representative result from three donors is shown.
(TIF)

S2 Fig Sorting strategy for LCs and BDCA3^+^ dermal DCs. (A) Epidermal cells isolated from abdominal skin were gated on DAPI^-^ cells then CD14^-^CD3^-^CD45^+^CD1a^+^ cells for sorting LCs. (B) Dermal cells isolated from abdominal skin were gated on live cells using forward and side scatter then on HLA-DR^+^BDCA3^+^ cells to sort BDCA3^+^ dermal DCs. Representative result from three donors is shown.
(TIF)

S3 Fig BDCA3^+^ and DC-SIGN^+^ cells separately stained in the dermis of inner foreskin explant tissues. Green: DC-SIGN^+^, red: BDCA3^+^, blue: DAPI. DC-SIGN^+^ dermal cells are smaller than BDCA3^+^ dermal DCs which are often found in clusters. The right panel shows the particular pattern of BDCA3^+^ dermal DCs in human foreskin. D: dermis. Scale bar indicates 15 μm. Representative result from three donors is shown.
(TIF)

S4 Fig DC migration assay using inner foreskin explants with or without allogeneic PBMC. (A) Scheme of procedure; Inner foreskin tissues were placed in the upper chamber of 24 transwell plates having 5 μm pore sized membrane. Medium or v-UL37GFP was placed inside the cloning cylinder and incubated for 72 hr. (B) Flow cytometric results after the culture; cells in the bottom chambers were collected and labelled for flow cytometry to enumerate and phenotype the cells which migrated out of the skin. Without PBMC, emigrated cells were rarely detected. Representative result from three donors is shown.
(TIF)
