# Supplementary figures and images for: Relay of Herpes Simplex Virus between Langerhans Cells and Dermal Dendritic Cells in Human Skin
Source: PLoS Pathog. 2015 Apr 13;11(4):e1004812. doi: 10.1371/journal.ppat.1004812 (PMC4395118; doi:10.1371/journal.ppat.1004812)

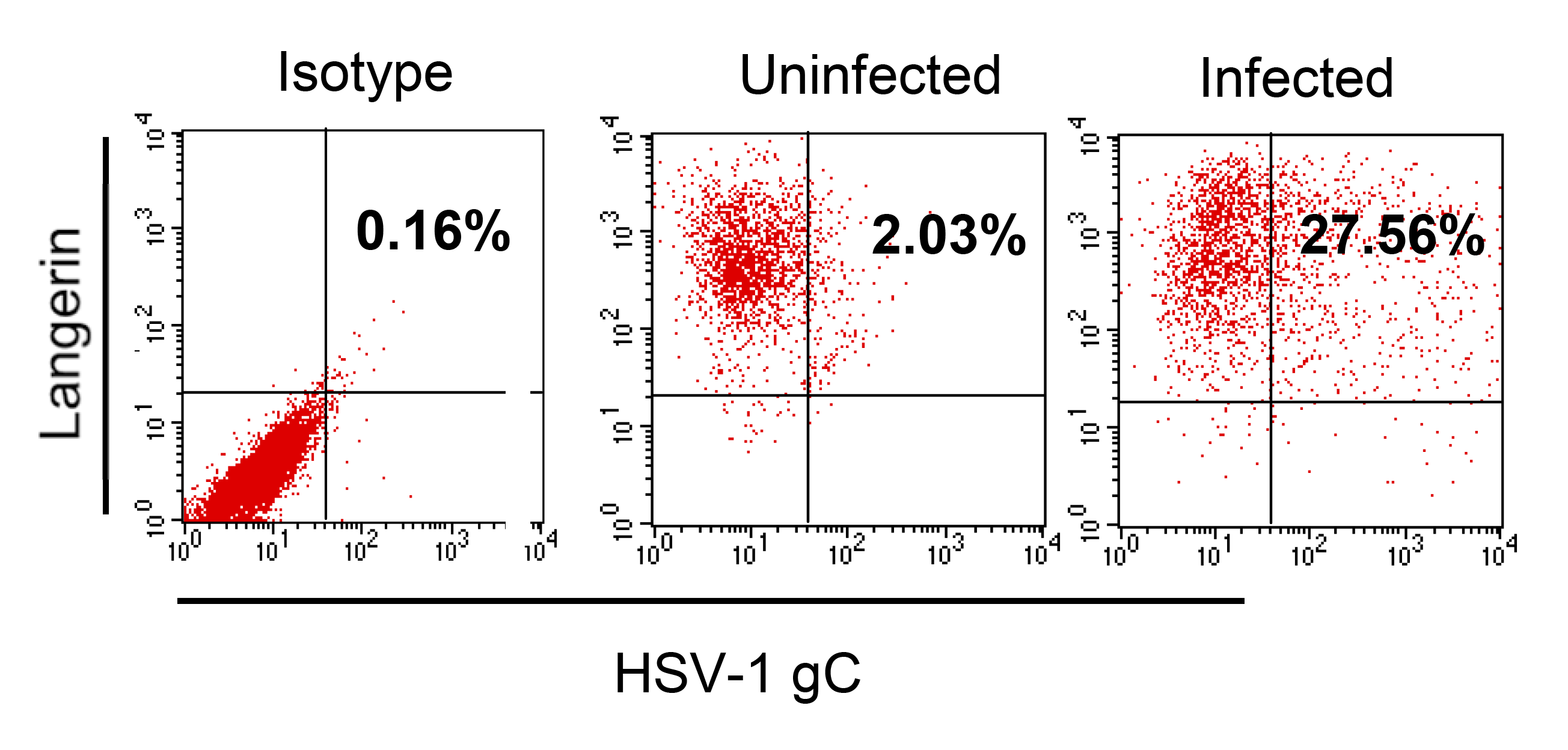

Supplement: S1 Fig — Epidermal sheets obtained from apronectomy were infected with HSV-1 (strain F) at an MOI of 5 for 2 hours at 37°C to allow virus penetration. After the incubation period, epidermal sheets were washed three times in PBS and then cultured for 24–48 hours in RPMI 1640 supplemented with 10% FBS and 25 μg/ml of gentamicin. Cells crawling out from the epiderminal sheets were collected and stained with monoclonal antibodies against langerin (R & D), HLA-DR (BD Biosciences) and HSV-1 glycoprotein C (gC; ViroStat). 28% of the emigrating LCs expressed gC at 48 hr p.i. Representative result from three donors is shown. (TIF) [file ppat.1004812.s001.tif]

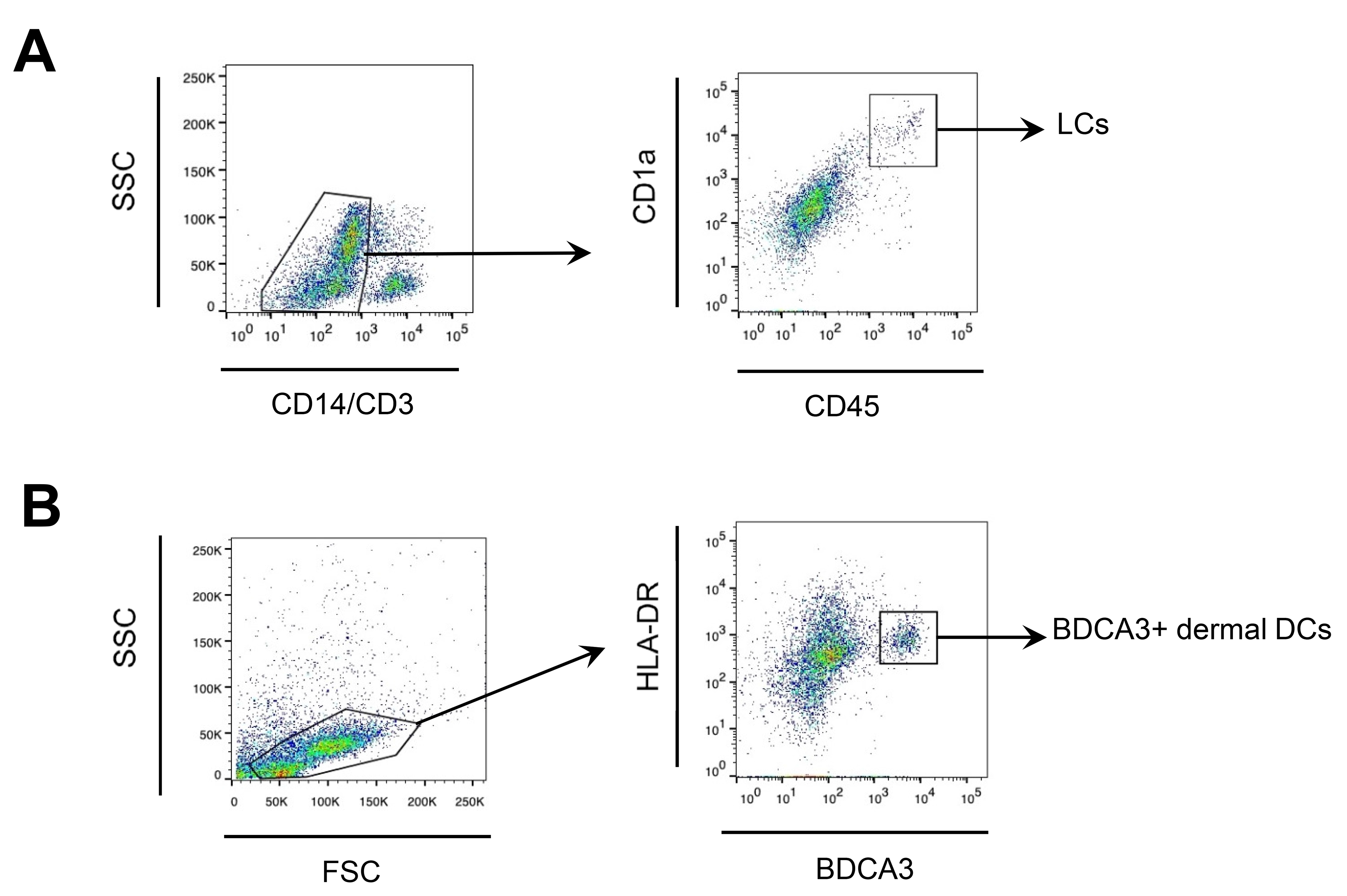

Supplement: S2 Fig — (A) Epidermal cells isolated from abdominal skin were gated on DAPI- cells then CD14-CD3-CD45+CD1a+ cells for sorting LCs. (B) Dermal cells isolated from abdominal skin were gated on live cells using forward and side scatter then on HLA-DR+BDCA3+ cells to sort BDCA3+ dermal DCs. Representative result from three donors is shown. (TIF) [file ppat.1004812.s002.tif]

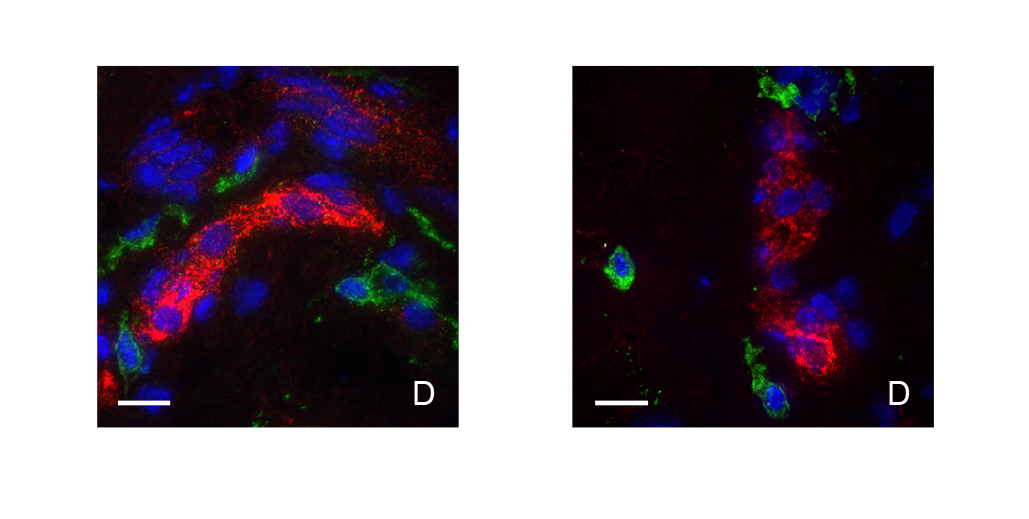

Supplement: S3 Fig — Green: DC-SIGN+, red: BDCA3+, blue: DAPI. DC-SIGN+ dermal cells are smaller than BDCA3+ dermal DCs which are often found in clusters. The right panel shows the particular pattern of BDCA3+ dermal DCs in human foreskin. D: dermis. Scale bar indicates 15 μm. Representative result from three donors is shown. (TIF) [file ppat.1004812.s003.tif]

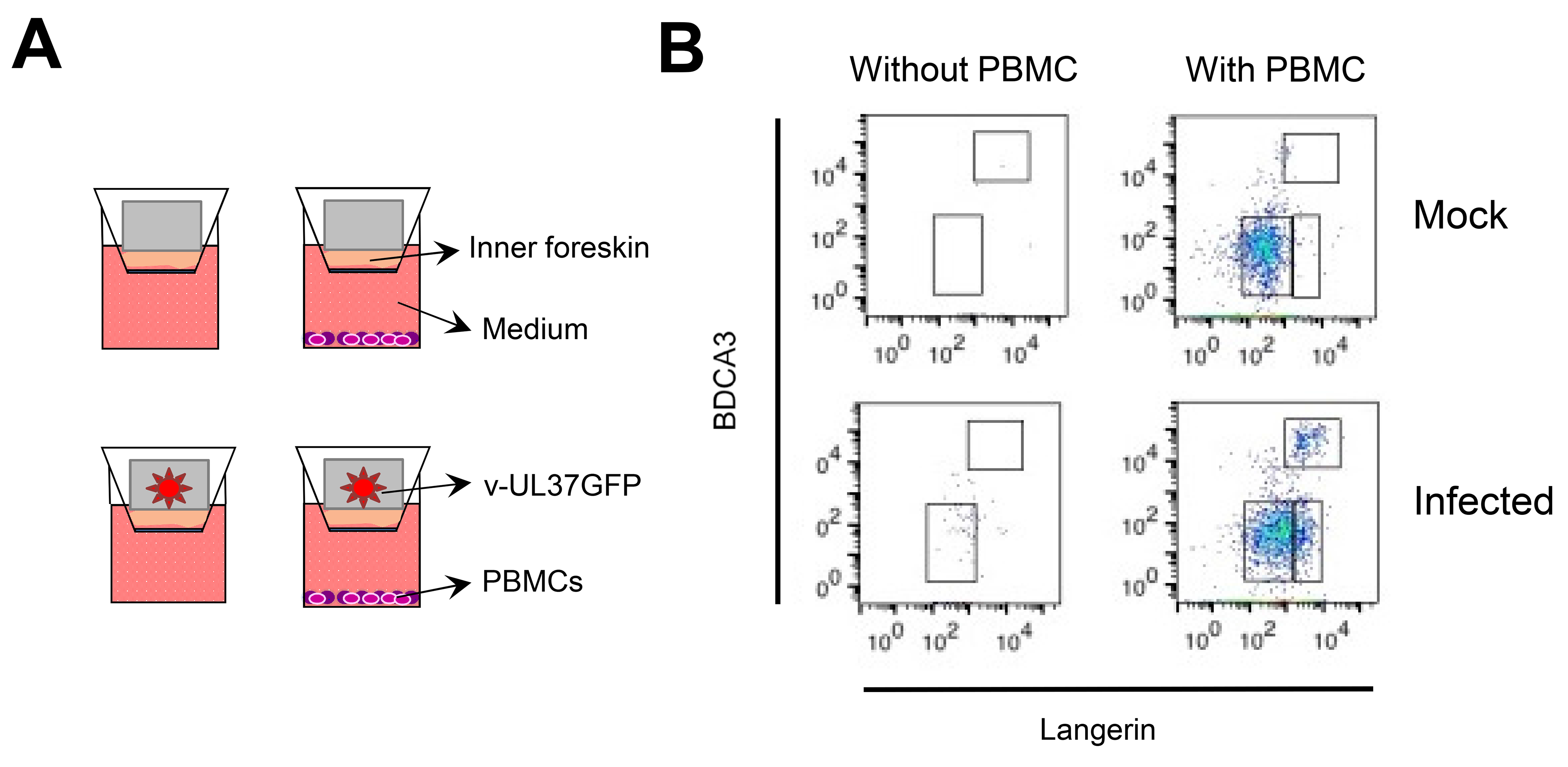

Supplement: S4 Fig — (A) Scheme of procedure; Inner foreskin tissues were placed in the upper chamber of 24 transwell plates having 5 μm pore sized membrane. Medium or v-UL37GFP was placed inside the cloning cylinder and incubated for 72 hr. (B) Flow cytometric results after the culture; cells in the bottom chambers were collected and labelled for flow cytometry to enumerate and phenotype the cells which migrated out of the skin. Without PBMC, emigrated cells were rarely detected. Representative result from three donors is shown. (TIF) [file ppat.1004812.s004.tif]
